# The chemical, biological, radiological and nuclear (CBRN) chain of survival: a new pragmatic and didactic tool used by Paris Fire Brigade

**DOI:** 10.1186/s13054-019-2364-2

**Published:** 2019-02-26

**Authors:** Franck Calamai, Clément Derkenne, Daniel Jost, Stéphane Travers, Isabelle Klein, Kilian Bertho, Frédéric Dorandeu, Michel Bignand, Bertrand Prunet

**Affiliations:** 1 Emergency Medical Department, Paris Fire Brigade, 1 Place Jules Renard, 75017 Paris, France; 2 Sudden Death Expertise Center, 75015 Paris, France; 3 Villacoublay Military Health Center, 78129 Velizy, France; 4 Val-de-Grâce Military Health Academy, 1 Place Alphonse Laveran, 75005 Paris, France; 5 French Armed Biomedical Research Institute, 91220 Brétigny-sur-Orge, France

**Keywords:** CBRN hazard, Chain of survival, Cognitive tool

## Background

The chemical, biological, nuclear, and radiological (CBNR) risk is indisputable and constant, but real situations are rare [1, 2]. The objectives of the emergency services facing these situations are primarily tactical, whether on the field or at the hospital door, and include reducing the morbidity and mortality of those exposed and limiting the risk for workers and care structures [3]. Their success meets the strategic and national objectives to minimize people’s panic and, as a result, the political effects. One of the challenges of emergency system personnel, who are the first responders, is to maintain the high level of performance required of pre-hospital and hospital workers, whether or not they are care providers [4]. However, effective preparation remains complex due to the low level of evidence to support tactical concepts, the pedagogical and logistical challenges of simulating the agents involved, and the scarcity of experts.

## The CBRN chain of survival

**Fig. 1:**
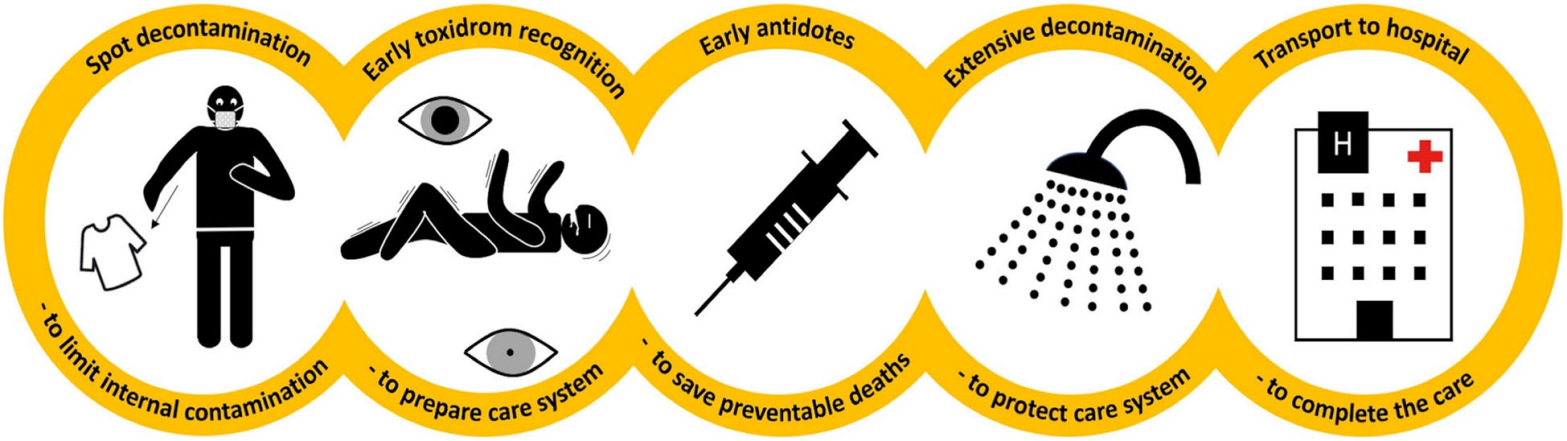
Sequence of events in CBRN situations is displayed schematically by “CBRN chain of survival” metaphor

The Paris Fire Brigade is confronted with these challenges at different levels of intervention, including the Primary Security and Answer Point, Basic and Advanced Life Support, and Emergency Medical and Crisis Dispatch, for an 800 km^2^ area and seven million inhabitants. Thus, the Paris Fire Brigade developed a cognitive tool to model the actions to be performed by first responders, so that simple and effective actions can be carried out in a precise order [5]. This article describes the survival chain for CBRN situations using analogies to existing survival chains (Fig. 1) [6, 7]. This chain is based on a known concept; therefore, we hope that its memorization will be easy and prolonged. Its yellow color is reminiscent of the classic CBRN logo. Like the survival chain of cardiac arrest, it is the weakest link in the chain that determines its overall robustness; thus, the first link is of predominant importance [8]. This chain could be used for public communication in time of crisis. Finally, this five-linked model provides a standard reference framework for both research and comparisons of practices in international healthcare systems. This is the first common basis for the creation of a register of contaminated persons. This current opinion describes the data supporting each link and recommends specific actions to strengthen the CBRN chain of survival.

The first link is the Spot Decontamination Link to prevent the spread of contamination [9, 10]. This link aims to reduce, as much as possible, the contamination present on the surface of victims to avoid the transfer of contamination to their internal environment through dermal or respiratory routes, or the transfer to teams and care facilities. It must be carried out as quickly as possible, given the rapid penetration of toxic substances. It is carried out by the patients themselves if they are able-bodied, or by rescuers when the victims are disabled. This decontamination involves the extraction of the contaminated area, the installation of an FFP2 respiratory mask, and the removal of the first layer of clothing. The exposed parts are decontaminated with a hyper-absorbent material or skin decontamination lotion.

Next is the Early Toxidrom Recognition Link to prepare care system. First, responders must quickly characterize chemical, radiological, or biological agents. The identification, even if supposed and unconfirmed, of an agent makes it possible to immediately address the emergency situation and to specify the nature of the risk to hospitals that will probably already be confronted with the spontaneous arrival of victims. Logistical (volume and nature of antidote), tactical (adaptation of protective clothing, persistence or not of the agent in question), and strategic (evacuation of areas) provisions depend on this link, for example, in a “powder envelop” situation [11, 12]. For chemical agents, this may involve searching for several symptoms in the same patient or for the same symptom in several patients. A disproportion between the high number of victims and the low power of an explosion, the presence of an “epileptic epidemic,” dyspnea, or ocular or neurological signs associated with toxidromes are highly suggestive [13]. For biological and radiological risks, and for some chemicals (e.g., Yperite), there are no early symptoms. Portable chemical, radiological, and biological detection devices exist, but are often imperfect [14]. For chemicals, the impurity of non-industrial products, their modification by the dissemination vector (e.g., explosion), and their association with other chemicals can make identification difficult; in the same way, odors traditionally attributed to chemicals are not reliable.

Next, the Early Antidotes Link can prevent deaths. Undressed victims are sorted to separate injured or symptomatic victims from others. The administration of specific antidotes (e.g., atropine, hydroxocobalamin) should not delay the symptomatic treatment of distress [15]. In cases of radiological and biological risk, except toxins, antidotes may exist but their use may be deferred until advised by an expert.

The Extensive Decontamination Link protects the healthcare system. The use of thorough decontamination lines (water and detergent) is only necessary for solid or liquid agents. Due to the long implementation time (often over 60 min), they contribute very little to improving the individual prognosis of victims. Despite often contrary beliefs by first responders and the public, they are only of real interest to protect hospitals from possible contamination transfer. If the supposed agent is non-persistent (gaseous), it will be possible to stay at this link and accelerate the evacuation of victims to hospitals. After thorough decontamination, victims are considered non-contaminating and can be treated without any specific protective measures for the caregivers.

The Evacuation to the Hospital Link completes the care. The decontamination of “chemical emergencies” takes precedence over medical and surgical emergencies, but this is not the case for radio-contaminated patients with critical medical and surgical conditions. After spot decontamination, they can be evacuated in extreme urgency using double packaging that limits the transfer of contamination to caregivers and hospital structures. Thorough decontamination will be carried out after medical and surgical survival procedures.

## Conclusion

The CBRN events are probably more of a means of disorganization and major terror than of mass destruction. During crisis situations, decision-making is most effective when the simplest and most effective ideas are used. The decreased cognitive performance of crisis room leaders and on-the-field emergency services induced by intense stress highlights the major importance of a “cognitive lifeline.” The CBRN survival chain is a didactic tool that supports the learning of five essential, inseparable, and ordered links in the care of victims in a CBRN situation.
